# Mandatory Reporting of Intimate Partner Violence: Examining Predictors and Experiences Among Intimate Partner Violence Victims

**DOI:** 10.1177/08862605251318273

**Published:** 2025-02-23

**Authors:** Astrid Gravdal Vølstad, Kevin S. Douglas, Solveig Karin Bø Vatnar

**Affiliations:** 1 Volda University College, Norway; 2 Simon Fraser University, Burnaby, BC, Canada; 3 Helse Bergen, Norway; 4 Oslo University Hospital, Norway; 5 Molde University College, Norway

**Keywords:** domestic violence, disclosure of domestic violence, legal intervention, intervention/treatment, battered women

## Abstract

Mandatory reporting of intimate partner violence (MR-IPV) is a controversial topic. This study examined the practice of MR-IPV by investigating what factors were associated with MR-IPV experience among victims of intimate partner violence (IPV). The study also investigated the experiences of IPV victims who have experienced MR-IPV, to better understand the consequences of MR-IPV. Eighty-six IPV victims were recruited through help services and administered a questionnaire about their experiences with IPV and MR-IPV. Multivariate logistic regression was used to explore statistical predictors of having experienced MR-IPV. Candidate predictors included IPV characteristics and risk factors, sociodemographic/contextual variables, and contact with the help services. IPV severity and persistence were of particular interest, as these define the threshold for whether MR-IPV applies in Norwegian law. IPV victims with MR-IPV experience were asked questions about the experienced consequences of MR-IPV. Neither characteristics of the IPV victimization, risk factors, sociodemographic variables nor contact with the help services were predictive of MR-IPV experience. However, having perpetrated severe psychological aggression was predictive of MR-IPV experience (OR = 4.99). Participants with MR-IPV experience (*n* = 39) reported both positive and negative consequences of MR-IPV, but generally more positive consequences for themselves. A majority agreed that, overall, they were better off after MR-IPV was used. Our results indicate that the Norwegian MR-IPV law might not be practiced as intended. The consequences of MR-IPV for IPV victims appear complex and warrant further study. However, overall, the use of MR-IPV led to positive reported consequences for the majority of the participants in this study.

## Introduction

Intimate partner violence (IPV) comprises physical and sexual violence, stalking, and psychological aggression (including coercive tactics) by a current or former intimate partner (Breiding et al., 2015). It is described by the World Health Organization (WHO) as a public health problem and has severe consequences both for the individuals involved and society at large (Lagdon et al., 2014; Pedersen et al., 2023; Stubbs & Szoeke, 2022; World Health Organization [WHO], 2017). To prevent IPV, many countries have adopted mandatory reporting (MR-IPV) laws that require a person with information about known or suspected IPV to report it (WHO, 2017). Who is obligated to report, what must be reported, and to whom a report is made varies between countries and states. For instance, in Kentucky, any person who has reasonable cause to suspect abuse, neglect, or exploitation must report to the Cabinet for Human Resources, which in turn must make a report and notify the police (Mandated Reporter Training, 2022). In California, MR-IPV only applies to health practitioners, who must report to the police if they treat a physical injury they suspect was caused by a firearm or assaultive or abusive conduct (Mandated Reporter Training, 2022). Other states, such as Alabama, do not have mandatory reporting laws for domestic violence (Mandated Reporter Training, 2022).

MR-IPV is controversial (Vatnar et al., 2021). Proponents argue that MR-IPV will help detect and prevent IPV, which could protect individuals subjected to IPV (hereafter called IPV victims) who may be unable to reach out for help. It may also improve responses from law enforcement and the criminal justice system and relieve IPV victims of the burden of having to report IPV themselves (Antle et al., 2010; Jordan & Pritchard, 2021). Critics argue that MR-IPV undermines the autonomy of IPV victims. They fear it will deter IPV victims from seeking help due to fear of retaliation from the partner, police involvement, or losing child custody. Critics hold that consent from the IPV victim must be secured before a report is made (Jordan & Pritchard, 2021; Lippy et al., 2020; Rodríguez et al., 2002; WHO, 2013).

The WHO does not recommend MR-IPV laws (WHO, 2013, 2017). However, a recent review of the MR-IPV literature did not find empirical support for this recommendation (Vatnar et al., 2021). The authors of the review found that there was a scarcity of research, and a moderate to high degree of bias in the research, as several studies systematically reported statistical minorities who opposed mandatory reporting as a more important finding than majorities who supported it. Further, the systematic review found that IPV victims generally were supportive of the law.

The research on MR-IPV that does exist has mainly focused on attitudes. Attitudes are important to consider, but there is no guarantee that they reflect reality. It is therefore crucial also to examine experiences; events that are lived through and not just imagined (APA, 2023). To our knowledge, only three studies of MR-IPV have included participants with experience of MR-IPV, leaving this area relatively unexplored (Antle et al., 2010; Jordan & Pritchard, 2021; Lippy et al., 2020). When studying participants with MR-IPV experience, it is also possible to investigate what factors are associated with experiencing MR-IPV, thus examining how the law transpires in actuality. Importantly, MR-IPV laws state thresholds for when the laws apply, but it remains unknown if these factors are important thresholds in practice.

In sum, MR-IPV is a controversial topic with a potentially high impact on the lives of IPV victims. Despite this, the literature is scarce and suffers from methodological issues. This study aims to add to the literature, examining statistical predictors of experiencing MR-IPV and the consequences of MR-IPV for IPV victims who have experienced it.^1^

### Previous Research on IPV Victims’ Experiences with Mandatory Reporting

As mentioned, few studies have examined experiences with MR-IPV. Among the few that have, Lippy et al. (2020) found that 8.2% of their 2,462 participants had experienced MR-IPV. When asked about their experiences, most participants reported that MR-IPV made their situation worse—either much worse (51.2%) or a little worse (11.9%). Follow-up questions revealed that the majority had negative experiences with the criminal legal system and child protective services. The study was set in the United States of America (USA) and recruited participants through a national domestic violence hotline. In contrast to the findings by Lippy et al. (2020), a qualitative study by Antle et al. (2010) found that 75% of 24 participants reported being better off after MR-IPV was used; only 1 person said she was worse off. The participants had both positive and negative experiences with MR-IPV. Half of the participants indicated making changes in their lives after the experience, and 54% indicated that they took steps to protect themselves. The study was set in Kentucky (USA).

Lastly, in another study set in Kentucky, Jordan and Pritchard (2021) asked 388 participants recruited from domestic violence shelters if foreknowledge about MR-IPV would have impacted their decision to seek help. A majority of the participants reported being less likely to seek help from a doctor or nurse (63.6%) or a therapist or counselor (59.7%) if they had known about MR-IPV. Less than half reported that foreknowledge would make them less likely to seek help from a domestic violence program (39.4%). The participants were not asked if the report impacted the IPV or the relationship with their partner, nor if they experienced MR-IPV as helpful. The study did not provide clear information on the prevalence of experienced MR-IPV among the participants.

The described studies investigated separate aspects of MR-IPV experience with different study designs and methodologies and found different results. Thus, the literature on the subject is both limited and inconclusive. No studies have been conducted outside the USA.

### Previous Research on Factors Influencing Mandatory Reporting Practices

A few studies have investigated which factors influence whether a mandatory report is made. Service providers report being less likely to use MR-IPV if they do not know how or to whom to report, if the IPV victim objects to the report, if they perceive that there is not enough evidence of the IPV, or if they believe the IPV is not severe enough (Cho et al., 2015; Rodriguez et al., 1999; Smith et al., 2008). Additionally, Rodriguez et al. (1999) found that physicians considered some factors indicative of always reporting: pregnancy, children or guns in the home, obvious injuries or repeated complaints of IPV, or immediate threats to a patient’s safety.

These studies rely on the service providers’ beliefs about their own behavior. Only one study has studied factors influencing MR-IPV use more directly. Lippy et al. (2020) used multivariate logistic regression to investigate if sexual orientation, gender identity, and race/ethnicity were predictors of having experienced MR-IPV; none were statistically significant.

### The Legal Context of the Current Study: The Norwegian MR-IPV Law

As mentioned, the content of MR-IPV laws varies significantly across countries. The present study was conducted in Norway, which has a section in the penal code termed “the duty to avert criminal offence” (The Penal Code, 2005, s. 196). The duty concerns several criminal offenses, including domestic violence (abuse in close relationships, s. 282) and severe domestic violence (aggravated abuse in close relationships, s. 283). These sections concern IPV that is “severe or persistent,” making these two factors’ thresholds for when MR-IPV applies. Any person who believes it to be certain or most likely that future severe or persistent IPV will occur has a duty to report it or seek to avert it “by other means.” Thus, there is a discretionary space in the law that allows for actions other than police reporting, as long as an alternative approach accomplishes the goal of averting the IPV. The duty is a part of the penal code and applies to any person, both in private and professional contexts, and applies regardless of professional confidentiality. This study examines MR-IPV in professional contexts only, including MR-IPV by both healthcare personnel and other help services such as domestic violence shelters. No studies have examined if the severity or persistence of the IPV are important thresholds for MR-IPV being used in practice.

Little is known about the practice of MR-IPV in Norway. The Commission on intimate partner homicide (IPH) in Norway found that there were several instances when laws and regulations, including MR-IPV, might not have been followed in cases that led to IPH (NOU, 2020, p. 17). Vatnar et al. (2022) examined the 1990 to 2020 IPH cohort in Norway (*n* = 224) and found that 70% of IPH victims and 80% of perpetrators had been in contact with the help services prior to the homicide, and there was prior IPV in 72% of IPH cases. However, information about the risk of IPV/IPH was forwarded to other professionals in only 21% of the cases where the victim had been in contact with professionals prior to the IPH. In cases where the perpetrator had been in contact with professionals, information about risk was forwarded in only 14.5% of the cases.

### Aims of the Current Study

The current study had two overall aims. The first was to examine the practice of MR-IPV by investigating what factors were associated with MR-IPV experience among IPV victims. Theoretically informed variables were chosen: IPV characteristics were of primary interest, in addition to sociodemographic and context variables. As evaluating the threshold for MR-IPV includes an element of risk assessment, we included IPV risk factors. The second aim was to examine the experiences of IPV victims who have experienced MR-IPV, to better understand the consequences of MR-IPV for IPV victims. We examined the following research questions:

1. To what extent are IPV characteristics and sociodemographic/contextual variables predictive of whether participants have experienced MR-IPV?
2. To what extent do IPV victims who have experienced MR-IPV report positive and negative consequences of MR-IPV for themselves and their partners?

## Method

The present study was part of the research project MANREPORT-IPV—a mixed methods study examining awareness of, attitudes toward, and experience with mandatory reporting of IPV. The study was approved by Oslo University Hospital’s Data Protection Official (22/00221). The Regional Committees for Medical Research Ethics considered the study to be health service research and thereby not within their mandate (257644).

### Participants

Of the 86 participants, most were female (85%). Most were native citizens—Norwegian citizens with no other citizenship (79%). Further, 10.5% were naturalized citizens (Norwegian citizens with immigrant background) and 9.3% foreign citizens (immigrants without Norwegian citizenship). Age ranged from 21 to 67 (*M* *=* 43.1, *SD* *=* 9.1). Education ranged from 6 to 25 years (*M* *=* 14.6, *SD* *=* 3.4). Half were employed (51.2%), 30.2% were receiving benefits from social security services, and 11.6% worked part-time while also receiving benefits from social security services. Most participants (83.7%) reported not currently having an intimate partner. Most participants had children (94%), either with their abusive partner (72.1%) and/or with other partners (32.6%). All participants had been in heterosexual relationships.

Most participants (95.3%) reported that they were not in an intimate relationship with IPV when they participated in the study. On average, the IPV relationship had lasted for 11.3 years (*SD* *=* 8.7, range = 0.25–33) and had ended 2.3 years ago (*SD* = 2.4, range = 0.07–14 years). However, many participants still had recent IPV experiences; 76.2% had been subjected to psychological IPV, 49.2% to physical IPV, 24.7% to sexual IPV, and 35.3% to severe IPV (weapons, strangulation, etc.) within the last year (measured by items modeled after the Spousal Assault Risk Assessment version 3; Kropp & Hart, 2015). Most participants had experienced IPV in only one relationship (68.6%), but some participants reported IPV in 2 (15.5%) or 3 (10.7%) relationships. Three participants (3.6%) reported no relationship with IPV, though they did report at least one form of IPV on subsequent behavioral measures. A little over a fifth of participants responded that they had also perpetrated IPV in 1 (19.8%) or 2 (2.3%) relationships.

### Procedures

Participants were recruited from 15 domestic violence shelters (69.8% of participants), 2 police districts (11.6%) and 15 alternative to violence (ATV) offices (18.6%). The ATV is a treatment service offering help to people who have perpetrated or been subjected to violence. The recruitment agencies were spread across all counties of Norway, both within rural and urban areas. Service providers received information about the research project and forwarded it to potential participants. If the IPV victim consented to participate, their contact information was given to the first author. The participants were not compensated for their participation. Data were collected between January 2022 and March 2023.

The participants could choose between filling out the questionnaire themselves (59.3%) or having it presented as a structured interview. Additionally, they could choose to meet the researcher digitally (66.3%) or in person. When meeting the researcher in person, structured interviews were performed in an office of the help service the participant was recruited from, unless the participant preferred another location. The questionnaire took on average 62 minutes (*SD* = 33 min) to complete for participants who chose self-report, and 112 minutes (*SD* = 53 min) for structured interviews. A professional interpreter was used when required (5.8%). A researcher was available for questions during data collection for all participants. The participants were encouraged to contact the help service they were recruited from if they experienced negative reactions after participating. They were also provided with the contact information of one of the project leaders, who is a clinical psychologist with significant clinical experience with and expertise in IPV. One participant contacted the project leader.

### Measures

Sociodemographic and contextual variables were drawn from previous IPV studies in Norway (e.g., Vatnar et al., 2017). Variables included recruitment source, gender, partner’s gender, age, partner’s age, the geographic region the participant lived in, origin, partner’s origin, number of children with their abusive partner and other partners, years of education, employment status, marital status, and a global question asking about the participants’ general health (ranging from 1—*very poor*, to 5—*very good*).

#### The Revised Conflict Tactics Scale

A Norwegian version of the Revised Conflict Tactics Scale (CTS2) was used to measure IPV experiences (Bendixen, 2005; Straus et al., 1996). The CTS2 is one of the most widely used measures of IPV and conflict behaviors in the general population and has demonstrated good validity and reliability (Chapman & Gillespie, 2019; Costa & Barros, 2016). It measures the number of times in the past year the participant and their partner engaged in different behaviors to deal with conflicts. As in the original CTS2, the response options for each item were *Never, Once, Twice, 3 to 5 times, 6 to 10 times, 11 to 20 times, More than 20 times*, and *Never in the past year, but it has occurred previously.* There are scores for negotiation (not used in this study), psychological aggression, physical assault, sexual coercion, and injury. Higher scores indicate several violent acts several times in the last year of the relationship. There are subscales for severity, distinguishing between “minor” and “severe” IPV. Additionally, scores can be calculated both as dichotomous (called “prevalence scores”) and as a scale measuring the number of incidents within the past year (called “chronicity scores”). In this study, both lifetime prevalence scores and past-year chronicity scores were used.

Due to the high prevalence of IPV victimization and low prevalence of IPV perpetration in the current sample, there was limited variation in some of the scales, particularly those measuring IPV perpetration (see Appendix A for the distributions). This concurs with Johnson (2006), who noted that samples from help services such as domestic violence shelters or the police have a higher likelihood of including participants who have experienced the Coercive Controlling Violence type from Johnson’s typology, which often involves more severe violence and less frequently involves bidirectional violence (Johnson, 1995; Kelly & Johnson, 2008). Cronbach’s alpha for the chronicity scales ranged from .038 to .965 and are presented in Appendix B. Several of the subscales measuring IPV perpetration and the subscale for minor psychological aggression victimization had Cronbach’s α < .600. We chose to include the CTS2 scales under the assumption that the low variance likely contributed to the low Cronbach’s alphas. While we cannot definitely attribute the Cronbach’s alphas to limited variance alone, the consistently high Cronbach’s alphas found in a number of other studies support the argument that the issue concerns limited variance, rather than the items failing to measure the same construct (Chapman & Gillespie, 2019; Costa & Barros, 2016). As the CTS2 prevalence scores are dichotomous variables, Cronbach’s alpha was not applicable. Perpetration of sexual coercion was not included in the analyses as no IPV victims reported perpetrating any item on the sexual coercion scale.

#### Accumulated Risk: Spousal Assault Risk Assessment Version 3

To measure risk factors for IPV, we developed a self-report checklist guided by the content of the Norwegian version of the Spousal Assault Risk Assessment version 3 (SARA-V3; Kropp & Hart, 2015; Vatnar et al., 2015). The SARA-V3 is a validated violence risk assessment instrument for IPV (Ryan, 2016). It contains 24 empirically demonstrated risk factors for IPV related to the IPV, the perpetrator, the victim, and the children (the latter not used in this study). The checklist developed for this study is intended for research purposes only and was developed with the intent of capturing a comprehensive set of IPV risk factors.

The SARA-V3 is a structured guide for practitioners performing risk assessment. Its 24 risk factors are operationally defined and intended to be rated by professionals based on their assessment (interviews; file reviews, etc.). We developed a single item, to be rated by participants, for each of the 24 SARA-V3 risk factors, using the SARA-V3 user guide definitions as guidance. An example item includes “Has the partner violence also included threats? Such as threats of murder, threats sent as a text message, statements such as ‘If you don’t. . . I will. . .’?’’ (The area “K2. Threats” from the SARA-V3 manual). The participants were asked to rate the occurrence of the items in the past year (“current” measure), as well as the time before (“previous” measure). The possible responses were *no* (coded as 0), *to some extent* (coded 1), *yes* (coded 2), and *unknown* (mean substitution used in cases where 25% or fewer of the items in a relevant subscale had *unknown* responses, see below). Scores were created by adding the items in a total score and subscales corresponding to the SARA-V3. The subscales consisted of risk factors related to the IPV (SARA-K; 8 items), the perpetrator/partner (SARA-U; 10 items), and the victim (SARA-O; 6 items). “Previous” scores were used for participants whose relationship ended more than 1 year ago, while “current” were used for the remaining participants.

To preserve as much data as possible, mean substitution was used for the SARA scores in cases when participants responded *unknown*, and specifications were met. The *unknown* score was replaced with the participant’s mean score on the relevant scale. This was only done for participants with *unknown* answers on 25% or fewer of the items in the relevant subscale. For instance, mean substitution was only used if participants had answered *unknown* on one or two items on the SARA-U (10 items in total). Participants with more *unknown* answers were collapsed with the missing category. Mean substitution was done in 47 cases for the SARA total score; 8 for SARA-K; 32 for SARA-U; and 3 for SARA-O. We note that 52.3% of participants responded *unknown* to one or more items in the SARA-U, introducing uncertainty in the measurement of this variable and the total risk score.

Cronbach’s alpha was not calculated for the SARA-V3 scores, as published guidance on the most appropriate indices of reliability and validity for risk assessment measures such as the SARA-V3 (i.e., structured professional judgment, or SPJ, measures) explicitly states that internal consistency is not recommended (Douglas et al., 2011). The SARA-V3 subscales are not intended to be an index of an underlying construct, but rather a compilation of risk factors associated with an external criterion (i.e., IPV). In principle, a set of largely uncorrelated items (with a very low alpha) could strongly predict an external criterion (IPV), if each were uniquely associated with that criterion.

#### Experience with Mandatory Reporting

Experience with MR-IPV was measured by asking the participants “Has someone from the help services used mandatory reporting regarding you and IPV with your consent?” Response options included *no, to some extent*, and *yes*. The participants were also asked if MR-IPV had been used *without* consent, with the same possible response options. Responding *yes* or *to some extent* on either MR-IPV with or without consent (or both) was considered indicative of MR-IPV experience. The participants with MR-IPV experience were asked follow-up questions about their experiences, rating agreement with different statements on a Likert scale ranging from 1 (*completely disagree*) to 4 (*completely agree*). Additional response options were *unsure* and *inapplicable*. The statements concerned the consequences of MR-IPV for themselves, their partner, and whether it impacted their interaction with the help services. The participants were also asked to briefly describe the positive and negative consequences of the MR-IPV in an open-ended response format. Additionally, they were asked “Do you wish someone from the help services had used mandatory reporting regarding you and IPV?” Possible answers were *no, to some extent*, and *yes*.

Prior to participation, the researchers briefly introduced the content of the MR-IPV law. Specifically, it was explained that if service providers believe severe IPV will occur, they might have a duty to avert it. The researchers also explained that MR-IPV applies even if the information the service providers are acting on is confidential. The law was not described in further detail.

### Statistical Analyses

Logistic regression was used to determine factors predictive of MR-IPV experience, with the outcome of MR-IPV experience coded as 0 = *no*, 1 = *yes*. Sample size and outliers were considered, and multicollinearity was checked by examining intercorrelations. The purposeful selection strategy recommended for small sample sizes was used (Altman, 1991; Hosmer & Lemeshow, 2012; Pallant, 2010). In step 1, we performed univariate logistic regression analyses with variables measuring the IPV victimization, characteristics of the IPV relationship, sociodemographic and contextual variables. As this study is exploratory and there is a limited understanding of the predictors of MR-IPV experience, we were liberal in the number of variables tested in univariate analyses.

In step 2, multivariate logistic regression models were tested based on the findings from the univariate models. We tested multivariate models for three categories of variables: (a) IPV victimization and characteristics of the IPV relationship (hereafter called IPV variables), (b) sociodemographic variables, and (c) contextual variables. We included statistically significant variables from the univariate analyses, as well as variables with *p* *<* .25. This threshold was chosen to ensure that no potential variables of interest were excluded, in accordance with the approach outlined by Hosmer and Lemeshow (2012). In step 3, a final multivariate model was created using the same inclusion criterion as in step 2 (*p* < .25). The Hosmer and Lemeshow test was used to examine goodness-of-fit for the models. Nagelkerke *R*^2^ and Cox and Snell *R*^2^ were used as measures of estimated explained variance.

In the analysis process, the variable measuring risk factors concerning the partner (SARA-U) and the number of children among participants with children met the threshold for inclusion in multivariate models but including them would mean losing statistical power in the models due to missing cases. Of these, the risk factor variable was considered particularly important theoretically, as the risk of future IPV is important when deciding if MR-IPV applies. We decided to run two sets of analyses: one including the risk factor variable and one without it. This was done to avoid excluding the variable entirely, in case there was a large enough effect that power would not be an issue.

## Results

### Prevalence of IPV and MR-IPV Experiences in the Sample

**Figure 1.**
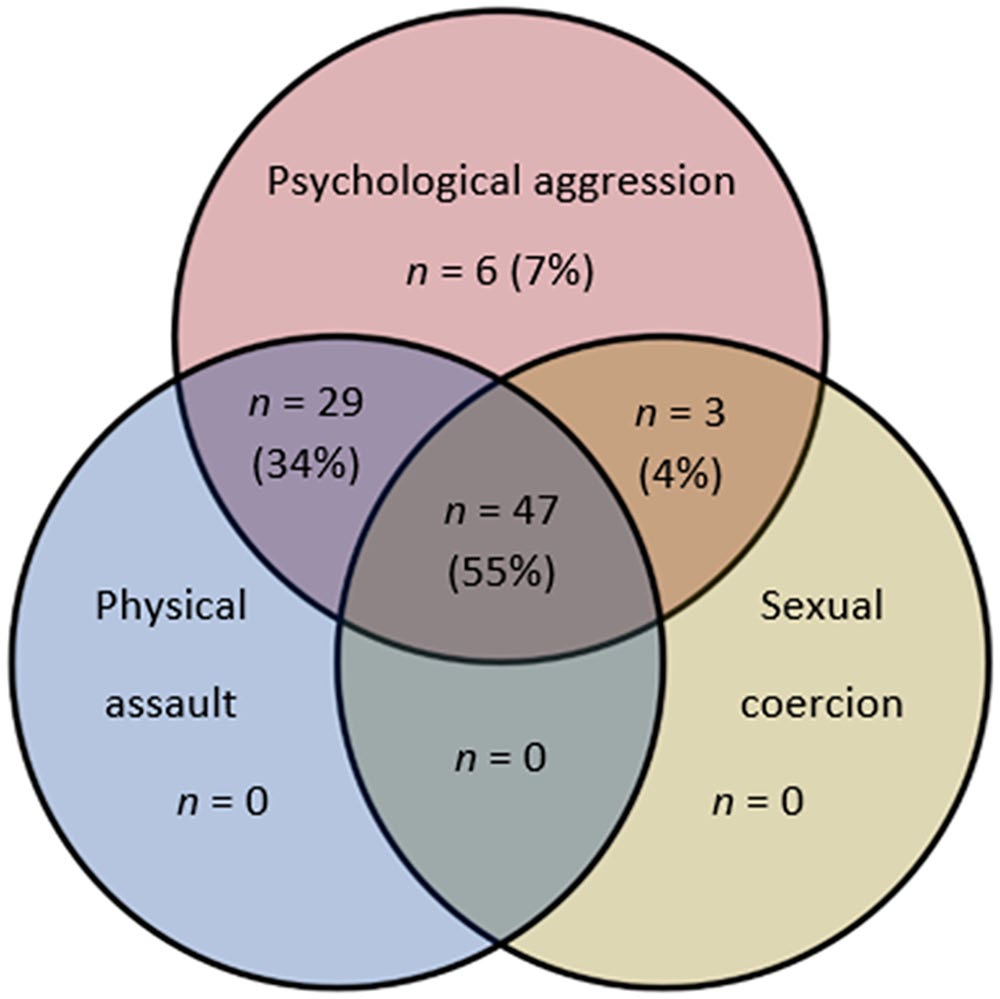
Prevalence of violence types the participants have been subjected to, measured by the CTS2 (lifetime prevalence). *Note.* Percentages refer to the percentage of the total sample (*n* = 86). CTS2 = Revised Conflict Tactics Scale.

Of the total sample (*N* = 86), 39 (45%) of the participants reported that service providers had used MR-IPV, either with their consent (*n* = 21), without consent (*n* = 6) or both (*n* = 12). Table 1 and Figure 1 show descriptive statistics of the IPV experienced by the participants. As illustrated in Figure 1, most of the participants (93%) had experienced more than one form of IPV—the most prevalent being all three forms of IPV (55%). Most of the participants had been subjected to at least one instance of severe IPV as measured by the CTS2 scales—psychological aggression (99%), physical assault (77%), sexual coercion (51%), and injury (59%).

**Table 1.** Descriptive Scores on the CTS2 for the Participants, Both Concerning Having Been Subjected to and Perpetrated IPV (*n* = 86).

Variable	Subjected to IPV						Perpetrated IPV					
	Prevalence		Chronicity				Prevalence		Chronicity			
	*n*	Frequency (%)	*n*	Mean (Range)	Median	*SD*	*n*	Frequency (%)	*n*	Mean (Range)	Median	*SD*
Psychological aggression	86	86 (100)	83	95.0 (0–200)	95	43.4	86	77 (90)	83	17.4 (0–125)	8	22.5
Minor	86	86 (100)	84	59.9 (0–100)	58	24.6	85	77 (90)	84	13.9 (0–79)	8	16.4
Severe	86	85 (99)	85	35.1 (0–100)	29	24.7	85	37 (43)	85	3.3 (0–50)	0	8.9
Physical assault	86	77 (90)	85	50.8 (0–300)	13	72.7	86	32 (37)	86	3.3 (0–79)	0	10.4
Minor	86	75 (87)	85	30.9 (0–125)	13	37.9	86	30 (35)	86	2.5 (0–54)	0	7.4
Severe	86	66 (77)	86	20.2 (0–175)	4	37.6	86	12 (14)	86	0.8 (0–25)	0	3.8
Sexual coercion	85	50 (58)	85	15.6 (0–75)	1	24.5	85	0	85	0.0	0	—
Minor	85	47 (55)	85	6.2 (0–25)	1	9.4	85	0	85	0.0	0	—
Severe	85	44 (51)	85	9.4 (0–50)	0	16.5	85	0	85	0.0	0	—
Injury	86	63 (73)	86	16.2 (0–127)	3	27.5	86	9 (11)	85	0.2 (0–6)	0	0.8
Minor	86	61 (71)	86	10.7 (0–50)	2	16.6	86	8 (9)	85	0.1 (0–2)	0	0.4
Severe	86	51 (59)	86	5.5 (0–77)	1	12.7	86	2 (2)	86	0.1 (0–6)	0	0.7

*Note.* Prevalence scores are dichotomous and indicate participants having experienced at least one item in the given subscale once in their life. Percentages refer to the percentage of the total sample (*n* = 86). The chronicity score measures the number of times within the last year of the relationship the participant or their partner engaged in different IPV behaviors. CTS2 = Revised Conflict Tactics Scale.

### IPV Characteristics, Sociodemographic, and Contextual Variables as Statistical Predictors of MR-IPV Experience

Logistic regression analyses are presented in Tables 2 to 5. In the initial univariate analyses, no variables measuring IPV victimization were predictive of MR-IPV experience. However, having *perpetrated* IPV was predictive of MR-IPV experience—specifically the following variables: the number of relationships where the IPV victims had perpetrated IPV (OR *=* 3.32); having perpetrated severe psychological aggression (lifetime prevalence; OR *=* 4.06); having perpetrated physical assault (lifetime prevalence, total score; OR *=* 3.07); and having perpetrated minor physical assault (lifetime prevalence; OR *=* 2.50). Additionally, living in the northern part of the country was predictive of *not* having experienced MR-IPV (OR = 0.21).

**Table 2.** Univariate and Multivariate Logistic Regression Analyses of MR-IPV Experience: IPV Variables.

Variable	Univariate Analyses			Multivariate Model (*n* = 78)		Multivariate Model Incl. SARA-U (*n* = 65)	
	*n*	OR	*p*	OR	*p*	OR	*p*
Types of IPV subjected to (psychological aggression)	85		.264		.697		.726
Psychological aggression and physical assault		7.083 (0.731–68.607)	.091	1.935 (0.100–37.553)	.663	0.550 (0.020–15.014)	.723
Psychological aggression and sexual coercion		2.500 (0.100–62.605)	.577	1.942 (0.057–66.643)	.713	1.203 (0.027–53.847)	.924
Psychological aggression, physical assault and sexual coercion		3.704 (0.401–34.222)	.248	0.951 (0.047–19.243)	.974	0.290 (0.010–8.538)	.473
Degree of perpetrated severe psychological aggression (CTS2 chronicity score, severe subscale)^a^	85	1.046 (0.981–1.114)	.167				
Having been subjected to physical assault (CTS2 prevalence score, total score)^a^	86	3.237 (0.632–16.587)	.159				
Having been subjected to severe physical assault (CTS2 prevalence score, severe subscale)	86	2.333 (0.799–6.811)	.121	0.767 (0.132–4.451)	.767	1.260 (0.157–10.113)	.828
Having perpetrated severe psychological aggression (CTS2 prevalence score, severe subscale)	85	4.062 (1.635–10.090)	.003	3.614 (1.174–11.125)	.025	3.349 (0.992–11.310)	.052
Having perpetrated physical assault (CTS2 prevalence score, total score)	86	3.070 (1.239–7.611)	.015	1.179 (0.357–3.898)	.787	1.501 (0.381–5.909)	.561
Having perpetrated minor physical assault (CTS2 prevalence score, minor subscale)^a^	86	2.500 (1.007–6.204)	.048				
Risk factors concerning the partner (SARA-U)	72	1.095 (0.970–1.237)	.144			1.141 (0.959–1.358)	.137
Risk factors concerning the IPV (SARA-K)	81	1.142 (0.997–1.308)	.055	1.157 (0.947–1.415)	.154	1.167 (0.915–1.488)	.213
Time since the IPV relationship ended	83	0.814 (0.655–1.012)	.064	0.859 (0.660–1.117)	.256	0.725 (0.512–1.027)	.070
Number of IPV relationships where the participant has perpetrated IPV	83	3.319 (1.197–9.202)	.021	1.652 (0.497–6.495)	.413	1.082 (0.285–4.103)	.908

*Note.* Regression models predicting MR-IPV experience (0 = no experience, 1 = experience). Model 1: Hosmer and Lemeshow test non-significant (*p* *=* .830). Nagelkerke *R*^2^ = .271, Cox and Snell *R*^2^ = .203. Model including SARA-U: Hosmer and Lemeshow test non-significant (*p* *=* .768). Nagelkerke *R*^2^ = .349, Cox and Snell *R*^2^ = .260. Reference categories for categorical variables are noted in parentheses. Variables tested in univariate analyses but not included in the table due to *p* > .25: currently being in an IPV relationship, duration of the IPV relationship, number of relationships where the participant has been subjected to IPV, total accumulated risk (SARA total score), risk factors concerning the participant (SARA-O). Additionally, all CTS2 subscales were tested in univariate analyses—both chronicity and prevalence scores for having been subjected to and perpetrated IPV. Both total scores and severity subscales were tested. Only the variables with statistically significant (*p* *<* .05) or trend significant (*p* *<* .25) results are included in the table. CTS2 = The Revised Conflict Tactics Scale; MR-IPV = mandatory reporting of intimate partner violence; SARA = Spousal Assault Risk Assessment.

a Variables not included in the multivariate model, as they overlapped with other variables that were included.

**Table 3.** Univariate and Multivariate Logistic Regression Analyses of MR-IPV Experience: Sociodemographic Variables.

Variable	Univariate Analyses			Multivariate Model (*n* = 83)	
	*n*	OR	*p*	OR	*p*
Number of children with abusive partner (among participants with children)^a^	62	0.578 (0.313–1.069)	.081		
Years of education	83	1.091 (0.956–1.245)	.198	1.083 (0.935–1.254)	.290
Marital status (not in a relationship)	86		.076		.141
In a relationship		0.311 (0.085–1.133)	.077	0.247 (0.058–1.046)	.058
Recent breakup/separation		0.389 (0.140–1.081)	.070	0.565 (0.182–1.759)	.325
Health (poor/very poor)	86		.252		.359
Neither good nor poor		0.733 (0.250–2.147)	.571	0.777 (0.236–2.558)	.678
Good/very good		0.412 (0.140–1.206)	.106	0.426 (0.124–1.457)	.174

*Note.* Regression models predicting MR-IPV experience (0 = no experience, 1 = experience). Multivariate model statistics: Hosmer and Lemeshow test non-significant (*p* *=* .152). Nagelkerke *R*^2^ = .146, Cox and Snell *R*^2^ = .109. Reference categories for categorical variables are noted in parentheses. Variables tested in univariate analyses but not included in the table due to *p* > .25: gender, age, partner’s age, origin, partner’s origin, having children, number of children with other partners, number of children the partner has with others, employment status. MR-IPV = mandatory reporting of intimate partner violence.

a Number of children was not included in the multivariate model due to loss of participants without children, and the subsequent loss of statistical power.

**Table 4.** Univariate and Multivariate Logistic Regression Analyses of MR-IPV Experience: Contextual Variables.

Variable	Univariate Analyses			Multivariate Model (*n* = 86)	
	*n*	OR	*p*	OR	*p*
Recruitment source (shelter)	86		.092		.148
ATV		0.381 (0.110–1.316)	.127	0.308 (0.075–1.263)	.102
Police		2.667 (0.629–11.306)	.183	2.107 (0.458–9.706)	.339
Geographic region (South-East)	86		.178		.262
South-West		0.643 (0.201–2.049)	.455	0.855 (0.249–2.934)	.804
Middle/Central		0.708 (0.203–2.467)	.588	1.444 (0.329–2.934)	.626
North		0.207 (0.051–0.840)	.028	0.268 (0.062–1.154)	.077

*Note.* Regression models predicting MR-IPV experience (0 = no experience, 1 = experience). Multivariate model statistics: Hosmer and Lemeshow test non-significant (*p* *=* .856). Nagelkerke *R*^2^ = .147, Cox and Snell *R*^2^ = .110. Reference categories for categorical variables are noted in parentheses. Number of help services contacted was tested, but not included in the table due to *p* > .25. Contact with specific health services was tested but not included in the multivariate models or the table to reduce the number of variables, as none were statistically significant at the *p* *<* .05 level. Specific help services contacted include shelter, family services, lawyer, police, doctor, emergency room, dentist, nurse, child and adolescent psychiatry, psychologist, child protective services, children’s house, school, and sexual assault center. MR-IPV = mandatory reporting of intimate partner violence; ATV = alternative to violence.

**Table 5.** Final Multivariate Logistic Regression Models for MR-IPV Experience.

Variable	Multivariate Model (*n* = 80)		Multivariate Model Incl. SARA-U (*n* = 66)	
	OR	*p*	OR	*p*
Perpetrating severe psychological aggression	4.994 (1.592–15.665)	.006	6.014 (1.286–28.132)	.023
Risk factors concerning the IPV (SARA-K)	1.145 (0.965–1.359)	.121	1.198 (0.924–1.552)	.172
Risk factors concerning the partner (SARA-U)			1.050 (0.841–1.311)	.665
Time since IPV relationship ended			0.614 (0.389–0.986)	.036
Marital status (not in a relationship)		.388		.176
In a relationship	0.433 (0.091–2.064)	.294	0.284 (0.028–2.909)	.289
Recent breakup/separation	0.435 (0.104–1.827)	.256	0.156 (0.020–1.218)	.076
Health (poor/very poor)		.428		.525
Neither good nor poor	0.783 (0.188–3.254)	.736	0.335 (0.050–2.235)	.259
Good/very good	0.385 (0.084–1.767)	.220	0.564 (0.086–3.703)	.551
Geographic region (South-East)		.519		.149
South-West	0.783 (0.170–3.599)	.753	0.183 (0.022–1.545)	.119
Middle/Central	1.488 (0.256–8.643)	.658	0.828 (0.096–7.121)	.864
North	0.337 (0.060–1.877)	.214	0.107 (0.011–1.002)	.050
Recruitment source (shelter)		.598		.644
ATV	0.469 (0.091–2.425)	.367	0.461 (0.056–3.806)	.473
Police	1.379 (0.177–10.728)	.759	0.385 (0.031–4.845)	.460

*Note.* Regression models predicting MR-IPV experience (0 = no experience, 1 = experience). Model 1: Hosmer and Lemeshow test non-significant (*p* *=* .972). Nagelkerke *R*^2^ = .376, Cox and Snell *R*^2^ = .281. Model including SARA-U: Hosmer and Lemeshow test non-significant (*p* *=* .964). Nagelkerke *R*^2^ = .536, Cox and Snell *R*^2^ = .399. Reference categories for categorical variables are noted in parentheses. MR-IPV = mandatory reporting of intimate partner violence; ATV = alternative to violence; SARA = Spousal Assault Risk Assessment.

In step two of the analysis process, three areas were identified to inspect further in multivariate analyses. Separate multivariate logistic regression models were created for IPV variables (Table 2), sociodemographic variables (Table 3), and contextual variables (Table 4). The variables in these tables with *p* < .25 were included in step 3. In the final multivariate model (Table 5), only having perpetrated severe psychological aggression remained statistically significant as a predictor of MR-IPV experience (OR = 4.99), controlling for other IPV variables, sociodemographic, and contextual variables. This held true both for the sets of analyses with and without the variable measuring risk factors concerning the partner (SARA-U), indicating robustness in this finding. For the sets of analyses that included the risk factor variables, another variable from the second analysis step was included, as it satisfied the inclusion criterion of *p* < .25: the number of years since the IPV relationship ended. This was predictive of *not* having experienced MR-IPV (OR = 0.61). There were no other differences between the models in terms of statistically significant variables.

### IPV Victims’ Experiences with Mandatory Reporting

The 39 participants who had experienced MR-IPV were asked follow-up questions about their experiences (see Table 6). In general, the *completely agree/disagree* response options were used more frequently than *partly*, indicating that the participants seemed to have held strong views about their experiences. Overall, the participants seemed to have had positive experiences with MR-IPV, though many also reported negative consequences. Most participants reported that MR-IPV had not weakened their trust in the help services (64% vs. 18%; see Table 6) or their care provider (56% vs. 10%). Most agreed that the one who had received the report had followed up on it thoroughly (69% vs. 8%). More participants agreed than disagreed that there were positive consequences for themselves (62% vs. 18%), though almost as many agreed (42%) as disagreed (46%) that there were negative consequences as well. Despite experiencing both positive and negative consequences, the majority of the participants (64%) agreed that, overall, they were better off after MR-IPV was used. For the items concerning their partners, the participants generally reported more negative consequences and were more unsure (see Table 6). The participants generally disagreed that there were few consequences of the MR-IPV (57% vs. 26%) and were divided on the question of whether the consequences were temporary (33% disagreed, 28% agreed, 23% unsure).

**Table 6.** Consequences of Use of Mandatory Reporting for Participants with Experience with it (*n* = 39).

Item	*n*	Completely Disagree (%)	Partly Disagree (%)	Partly Agree (%)	Completely Agree (%)	Unsure (%)	Inapplicable (%)
The one who received the report followed up on it thoroughly	37	2 (5)	1 (3)	6 (15)	21 (54)	3 (8)	4 (10)
I got a less trustful relationship with my care provider	38	22 (56)	0	2 (5)	2 (5)	3 (8)	9 (23)
I got a less trustful relationship with the help services in general	38	22 (56)	3 (8)	1 (3)	6 (15)	3 (8)	3 (8)
It led to positive consequences for me	38	5 (13)	2 (5)	7 (18)	17 (44)	4 (10)	3 (8)
It led to positive consequences for my partner	38	10 (26)	1 (3)	5 (13)	8 (21)	11 (28)	3 (8)
It led to negative consequences for me	38	16 (41)	2 (5)	8 (21)	8 (21)	3 (8)	1 (3)
It led to negative consequences for my partner	38	5 (13)	1 (3)	4 (10)	12 (31)	11 (28)	5 (13)
Overall, I was better off	38	4 (10)	1 (3)	5 (13)	20 (51)	5 (13)	3 (8)
Overall, my partner was better off	38	11 (28)	3 (8)	1 (3)	1 (3)	18 (46)	4 (10)
There were few consequences for me	38	19 (49)	3 (8)	5 (13)	5 (13)	5 (13)	1 (3)
The consequences were temporary	38	13 (33)	0	6 (15)	5 (13)	9 (23)	5 (13)

*Note*. Percentages refer to the percentage of the sample of participants with experience with MR-IPV (*n* = 39). MR-IPV = mandatory reporting of intimate partner violence.

On the open-ended questions about *positive* consequences, the participants reported the following (in no particular order): the IPV stopped; the IPV victim got away from the IPV relationship; they felt more secure; there were positive consequences for their children; they received help from services such as domestic violence shelters; specific protection measures were provided (e.g., a restraining order or a personal attack alarm; the partner was prosecuted). They also reported that the interactions with the help services helped them understand the severity of the violence they had been subjected to, and empowered them to make pivotal changes, such as making plans for a possible crisis and leave the IPV partner. Two participants reported that there were no positive consequences. Of the 39 participants with MR-IPV experience, 33 responded to the open-ended question.

On the open-ended questions about *negative* consequences, the participants reported the following (in no particular order): continued threats, conflicts, and false accusations from the IPV partner; safety measures that interfered with daily life; negative consequences for the children or their relationship with their children; not being believed or fear of this; fear of negative consequences for the partner; fear of long-term consequences such as what would happen after the partner is released from jail; not being sufficiently followed up on after MR-IPV had been used; experiencing that the process in itself was taxing emotionally and frightening (though some added that it was worth it even though they had a negative experience at the time). Five participants stated that there were no negative consequences; 12 did not respond.

The participants were also asked whether they wished someone from the help services had used MR-IPV. Of the participants with MR-IPV experience, 21% responded *no*, 18% responded *to some extent*, and 51% responded *yes* (10% missing).

## Discussion

The aims of the study were to examine predictors of MR-IPV and to investigate the experienced consequences of MR-IPV. The main findings in our study were as follows: (a) neither characteristics of the IPV victimization, nor sociodemographic or contextual factors, were predictive of MR-IPV experience; (b) having perpetrated severe psychological aggression toward a partner was predictive of MR-IPV experience (OR = 4.99); (c) many participants had not experienced MR-IPV despite having experienced severe and persistent IPV; and (d) the participants tended to report positive consequences of MR-IPV, though many also reported negative ones. A majority (64%) reported that they were better off after MR-IPV was used. The participants were more unsure about and reported more negative consequences for their partners.

### Findings Concerning MR-IPV Practices

According to Norwegian law, MR-IPV applies when a person has reason to believe that persistent or severe IPV most likely will occur (The Penal Code, 2005, s. 196). Previous IPV is one of the best documented risk factors for future IPV (e.g., Kuijpers et al., 2011). It was therefore unexpected that characteristics of the IPV victimization were not predictive of MR-IPV experience. Caution should be taken when interpreting this finding. At first glance, it could suggest that IPV characteristics in practice are unimportant when evaluating whether MR-IPV applies. However, we cannot infer a decision-making process, only the outcome of either a decision-making process or the lack of one. It is unclear if our findings result from IPV severity not being used as a criterion for MR-IPV, if the participants’ situations were misjudged if there was low compliance with the law, or simply if the law’s requirement to act was unknown. In addition, there was a high prevalence of severe and persistent IPV in the sample. Limited variance likely was an issue for some of the scales, particularly the dichotomous prevalence scores with very high prevalence (e.g., severe psychological aggression with 99% endorsement). Moreover, from a clinical perspective, there might be a ceiling effect in the sense that once the IPV reaches a certain severity, clinicians might treat all cases as “severe IPV,” regardless of whether the IPV victim obtained a CTS2 severe physical assault score of 100 or 150. Thus, some of the variations in the CTS2 scales might have had limited clinical significance. However, regardless of why IPV characteristics were not predictive of MR-IPV, the finding suggests that the practice of MR-IPV might differ from what is outlined in the law.

Our results partly mirror findings from studies of service providers. The review by Vatnar et al. (2021) found that very few service providers had reported IPV in accordance with MR-IPV law. The professionals studied were physicians, dentists, nurses, emergency department staff, social workers, and psychologists. The studies were primarily conducted in the USA, though one study was set in South Korea (Cho et al., 2015). Two studies specifically point to IPV characteristics and having children in the home as important for the professionals’ decision to use MR-IPV (Cho et al., 2015; Rodriguez et al., 1999), which is in contrast to our findings that IPV victimization and having children were not predictors of MR-IPV experience. However, we note that most of the participants in our study had children (94%), and an effect would have to be very large to be detectable using our data. One possible interpretation is that the presence of children could prompt service providers to report to child welfare services. However, this does not necessarily lead to prevention of the IPV. One recent qualitative study found that child welfare workers considered the child their main responsibility, had limited knowledge of MR-IPV obligations concerning IPV between adults, and highlighted the adults’ autonomy and personal responsibility to seek help for the IPV (Dahl et al., 2024).

Our second main finding was that having *perpetrated* severe psychological aggression was a predictor of experiencing MR-IPV, both in the sets of analyses with and without the risk factors concerning the partner. This finding was unexpected, though there are some possible explanations. The psychological aggression can be viewed as a resistance strategy for dealing with IPV, or as bidirectional IPV, which are risk factors for recurring IPV and IPH, respectively (Goodman et al., 2005; Vatnar et al., 2017). Service providers could have recognized the added risk and used MR-IPV as a response. However, we would expect this to be reflected in an association between more severe IPV and MR-IPV experience, which we did not find. Another explanation could be that individuals who perpetrate psychological aggression against their partner might view the IPV as conflicts, not identify as victims of IPV, or be reluctant to leave their partner. Service providers might act differently toward individuals they perceive as unwilling to take steps to reduce the risk of further IPV themselves. This possibility illustrates that the context of the IPV might play an important role when service providers decide to use MR-IPV. The CTS2 has been criticized for not taking the context of the IPV into account (Jones et al., 2017). In our study, the variable measuring risk factors concerning IPV (SARA-K) was not statistically significant in the final multivariate model. However, we note that it was marginally significant in univariate analyses (*p* = .055), and there might have been an effect that was too small to detect with our sample size. Future studies should further examine whether particular elements of the overall IPV risk situation have an impact on MR-IPV experiences and decision-making among service providers.

In the sets of analyses where risk factors concerning the partner were included, the number of years since the IPV relationship ended was significantly predictive of not having MR-IPV experience. This might reflect the fact that MR-IPV has gained attention in Norway in recent years. However, the finding was less robust than others, as the variable was included in only one of the sets of analyses.

### IPV Victims’ Experiences with Mandatory Reporting of IPV

Of our 86 participants, 39 had experienced MR-IPV being used by a professional from the help services, either with (*n* = 33) or without (*n* = 18) their consent (12 participants reported both). Though we cannot draw conclusions about the rate of MR-IPV usage, an important finding was that more than half of our participants had *not* experienced MR-IPV despite having been subjected to severe and persistent IPV; all had experienced severe psychological aggression, 70% severe physical assault, 54% severe sexual coercion and 55% severe injury. This is a concern, given that a large percentage of IPV homicide victims are known to have experienced past serious or repeated IPV, both in Norway and in international studies (Campbell et al., 2007; Vatnar et al., 2017).

The participants reported both positive and negative consequences of MR-IPV, but generally more positive consequences. This highlights the complexity in the situations where MR-IPV applies; there might be both positive and negative short- and long-term consequences for the same individual, and it might also vary between individuals. The results mirror findings by Antle et al. (2010), who found that participants reported experiencing both positive and negative consequences of MR-IPV, but that most (75%) reported that they were better off after MR-IPV was used. In contrast, Lippy et al. (2020) found that most participants experienced negative consequences. A possible explanation of the differences is the timing and context of the participation. It might be that people view MR-IPV differently if they are acutely experiencing IPV, as was the case in Lippy et al.’s (2020) study.

Concerning the help services, most participants experienced that, if a report was made in the process of using MR-IPV, the one who received the report followed up on it thoroughly (69% agreed vs. 8% disagreed). Most also reported that MR-IPV did not weaken their trust in their care provider (56% vs. 10%) or the help services in general (66% vs. 18%). These findings are important considering the debate surrounding MR-IPV. Concerns have been raised that MR-IPV will weaken people’s trust in the help services and decrease their willingness to disclose IPV or seek medical care (Jordan & Pritchard, 2021; WHO, 2013). In this study, we did not find support for this argument. Our findings differ from those of Jordan and Pritchard (2021), who found that more than half of the participants would have been less likely to seek help from a doctor/nurse or counselor/therapist if they knew that the service provider would have to report the IPV.

When comparing results, it is important to keep in mind that MR-IPV law, healthcare systems, and criminal systems differ between countries. Experiences with and trust in the help services and law enforcement might differ between contexts and countries, affecting the MR-IPV experience. Additionally, the aim of the Norwegian law is averting the IPV, which can be accomplished without police reporting. Thus, as the laws differ in content, our results might not generalize to other countries. On the other hand, reporting to the police is explicitly stated in the law as a means to avert and might be needed to ensure safety. The criteria for MR-IPV are also relatively similar across countries, introducing the basic idea that the help services are mandated to intervene in some way when faced with cases of severe IPV.

### Limitations

Some limitations should be noted. First, we based the measurement of experience with MR-IPV on how the participants understand MR-IPV, which might be incorrect. However, as part of the MANREPORT-IPV study, the participants were asked to describe the mandatory reporting law. Most participants grasped the basic idea that MR-IPV is about averting severe IPV, harm, or criminal offense. If the participants asked what MR-IPV was, the researchers provided a brief description.

Second, we did not ask the participants who from the help services used MR-IPV. This would have been useful information, and future studies should include a measure of who used MR-IPV. Additionally, we did not have information about the timing of the MR-IPV experience. Although we had information about contact with the help services, we did not know the extent of contact with the help services *prior* to MR-IPV being used. This could be important to investigate in future studies.

Another limitation of the study is the sample size. The small sample size in the subgroups and conducting multivariate logistic regression analyses increases the probability of making a type II error, even if the number of univariate tests is high (which would increase the probability of a type I error). This is of particular importance to note given that our main finding is that we found no association between IPV victimization and MR-IPV experience. Related to this, some of the scales in the CTS2 had low Cronbach’s alpha. We decided to include the scales despite this, under the assumption that low variability among many item scores likely contributed to the low internal consistency scores. We note that we do not know that low variability is the only reason for low Cronbach’s alphas, and future research is needed to replicate the results. However, the issue with low Cronbach’s alpha was mostly the case for the scales measuring the participants’ IPV perpetration, and we did not find statistically significant effects of the CTS2 variables that did have high scores on internal consistency, suggesting that low reliability was not an overarching explanation for findings of non-association. We also note that low Cronbach’s alpha was not an issue with the variable that was statistically significant in the final multivariate model (CTS2 prevalence score for severe psychological aggression perpetration). The variable is dichotomous, and Cronbach’s alpha is thus inapplicable. It also had an endorsement rate of 43%, meaning that it was not hampered as a predictor variable by a low response rate.

Additionally, participants who were on good terms with the help services who recruited them might be more likely to participate, and probably also more likely to be asked to participate. We might not have included individuals who have had the most negative experiences with MR-IPV, as they might be more likely to have severed contact with the help services. Recruitment might also have been skewed toward recruiting individuals with experience with MR-IPV, as the service providers we collaborated with for recruitment knew the focus of the study.

We also note that the sample consisted of mostly Norwegian women without immigrant background who had been in a heterosexual relationship. The results might not generalize to populations with other characteristics. There were few men (15%), though our numbers were higher than those found in Norwegian domestic violence shelters (9%–10% men; Bufdir, 2023). The results are also not necessarily generalizable to all IPV victims, as this study only included individuals who had been in contact with the help services.

### Implications for Practice, Policy, and Research

Our main finding, that characteristics of the IPV victimization were not predictive of MR-IPV experience, suggests that the MR-IPV law might not be practiced as intended, although again we issue the caveat that some of those analyses might have been underpowered. There is a need for further research to determine if this is due to the law being unknown, or if IPV characteristics are not considered as important thresholds for MR-IPV use in practice. Our findings may reassure help service providers who hesitate to use MR-IPV for fear of negative consequences. However, it also highlights the complexity of situations involving IPV. Future research should dive deeper into the consequences of MR-IPV for IPV victims, as our results suggest that they may be complex. Additionally, it would be important to examine the experiences of individuals who are no longer in contact with help services, as these might differ from those in our sample. Lastly, this was an explorative study in a field with little existing research. Future research is needed to replicate the results and uncover the causal relationships at play.

## Supplemental Material

sj-docx-1-jiv-10.1177_08862605251318273 – Supplemental material for Mandatory Reporting of Intimate Partner Violence: Examining Predictors and Experiences Among Intimate Partner Violence Victims Supplemental material, sj-docx-1-jiv-10.1177_08862605251318273 for Mandatory Reporting of Intimate Partner Violence: Examining Predictors and Experiences Among Intimate Partner Violence Victims by Astrid Gravdal Vølstad, Kevin S. Douglas and Solveig Karin Bø Vatnar in Journal of Interpersonal Violence

## Notes

1. The term “predictor” is used in this article as a statistical term and is not meant to imply causality.
