# Supplementary material for: Mandatory Reporting of Intimate Partner Violence: Examining Predictors and Experiences Among Intimate Partner Violence Victims
Source: J Interpers Violence. 2025 Feb 23;41(3-4):889–917. doi: 10.1177/08862605251318273 (PMC12743134; doi:10.1177/08862605251318273)
Supplement: sj-docx-1-jiv-10.1177_08862605251318273 – Supplemental material for Mandatory Reporting of Intimate Partner Violence: Examining Predictors and Experiences Among Intimate Partner Violence Victims [file sj-docx-1-jiv-10.1177_08862605251318273.docx]

**Appendix A**

**Distributions of the CTS2 Scales With Cronbach’s α < .600**

The histograms in Figure B1 present the distributions of the responses on the scales that had a Cronbach’s α < .600. The scales measuring minor psychological aggression victimization and perpetration had more variation in the overall scores than the remaining scales. We therefore present the scores on each item for these scales as well for these scales in Figure B2 and B3.

**Figure A1**

*Responses on the CTS2 scales with Cronbach’s α < .600*


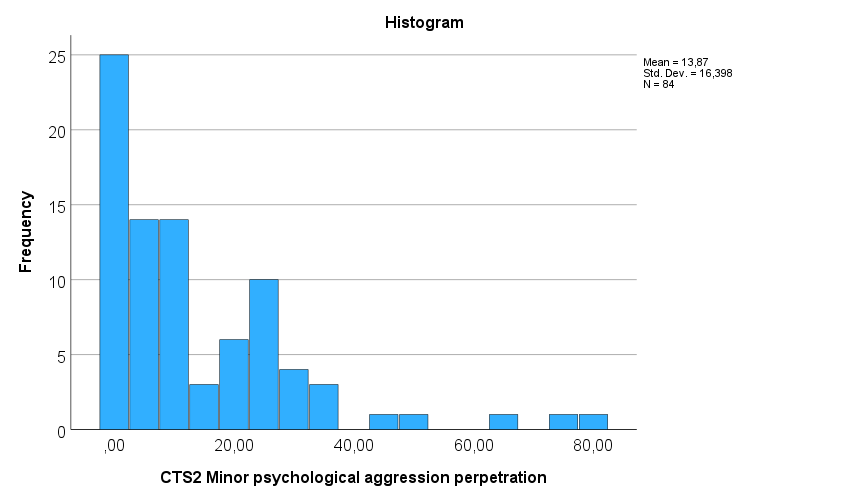

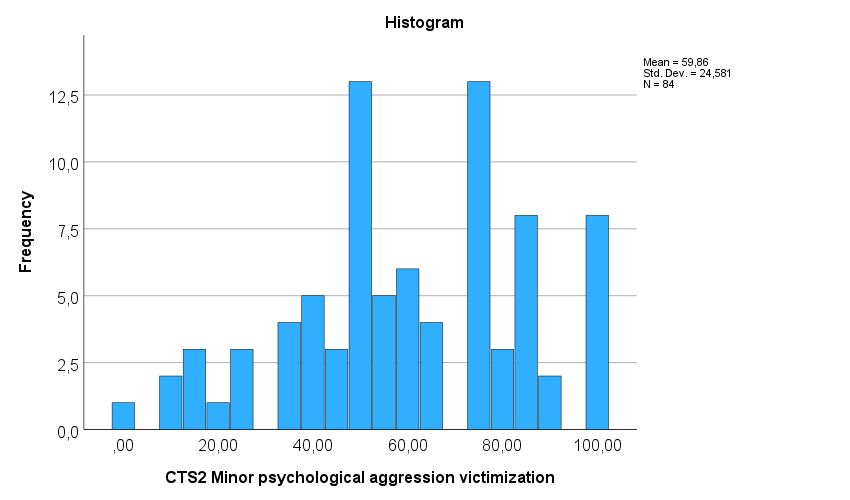


**Figure A1 (continued)**


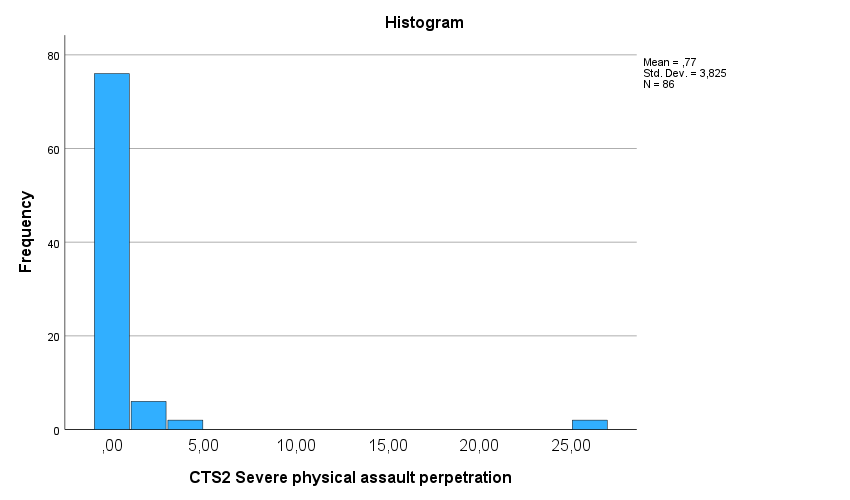

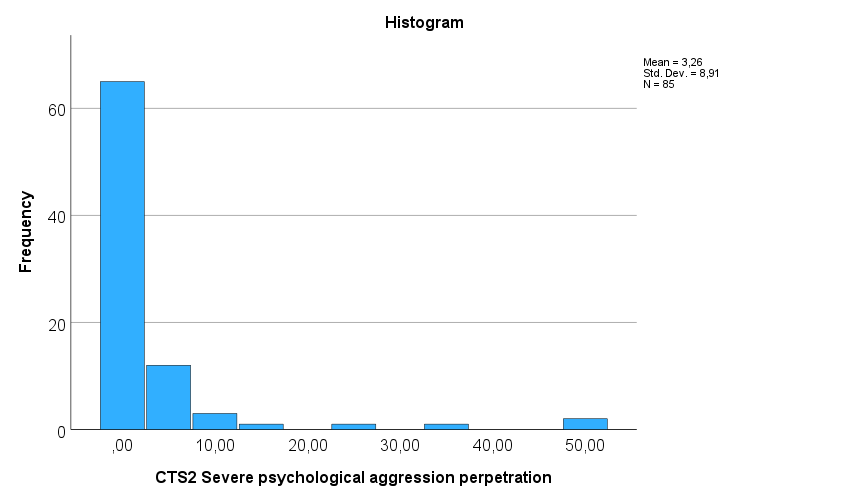


**Figure B1 (continued)**


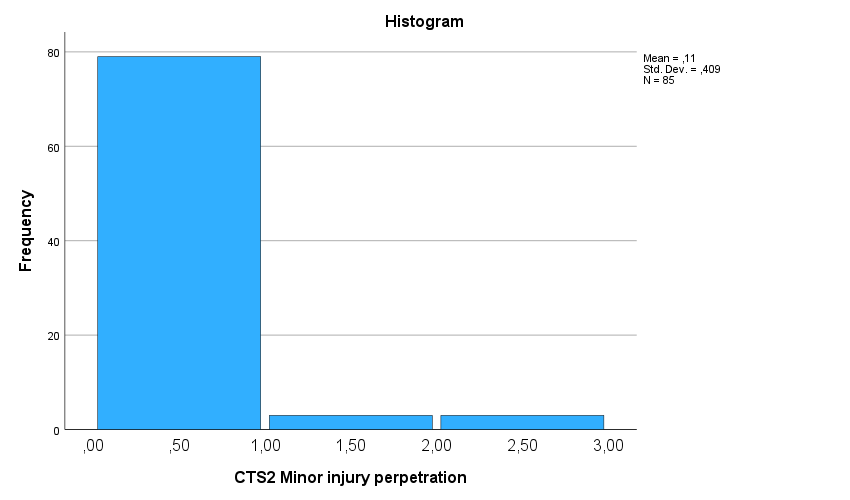


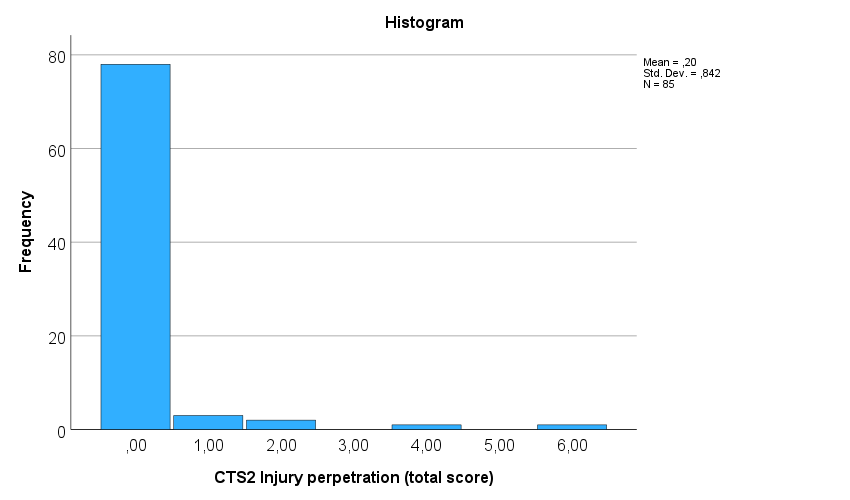


**Figure A2**

*Distribution of Items in the CTS2 Subscale Minor Psychological Aggression Victimization*


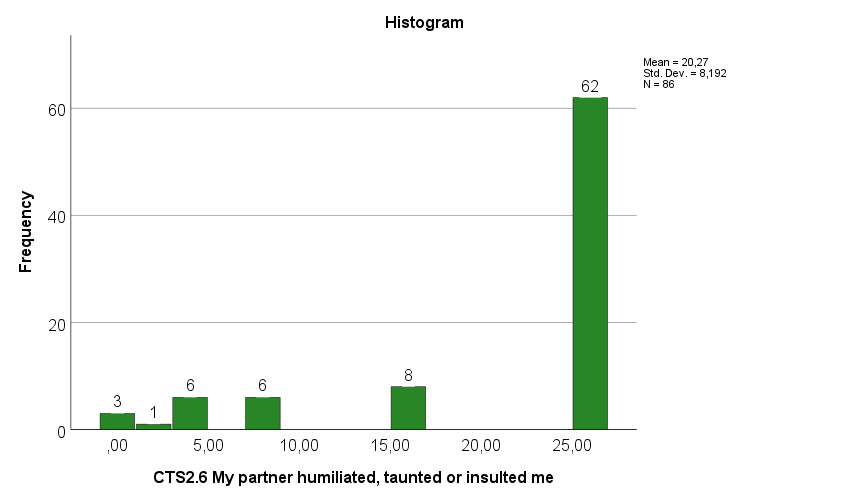

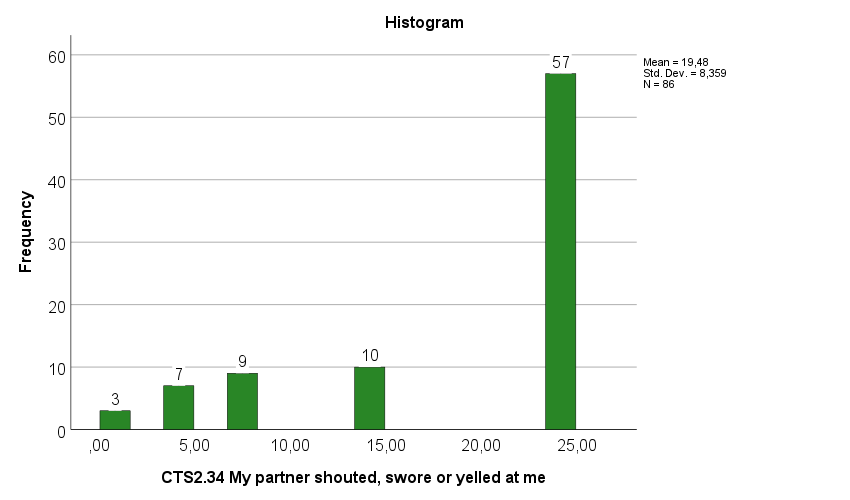


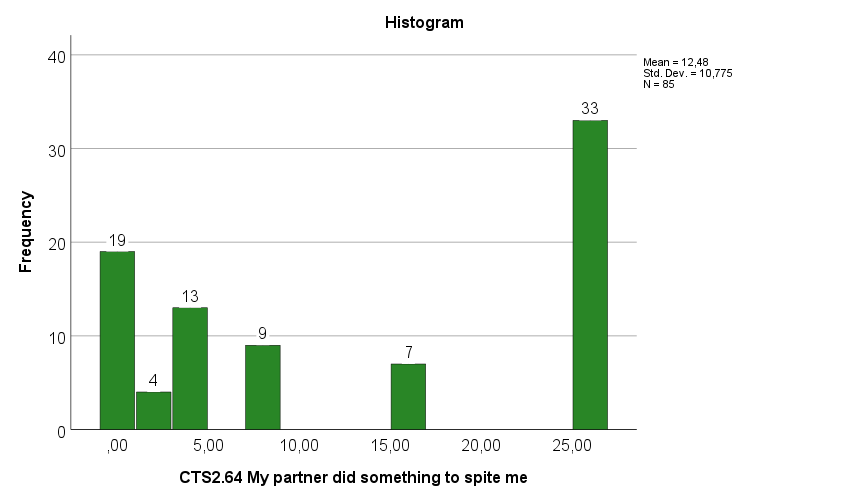

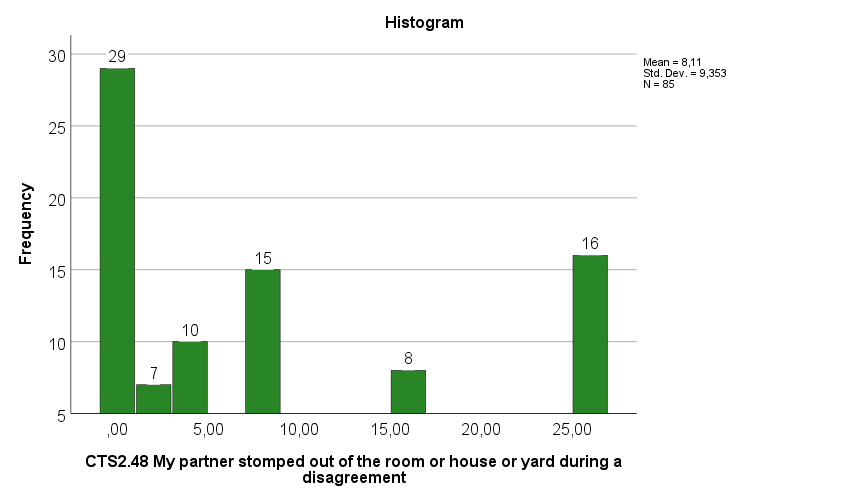


**Figure A3**

*Distribution of Items in the CTS2 Subscale Minor Psychological Aggression Perpetration*


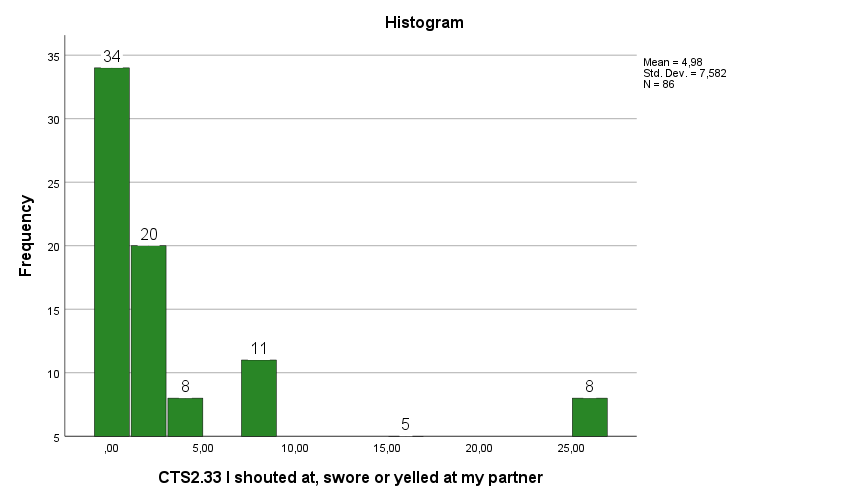

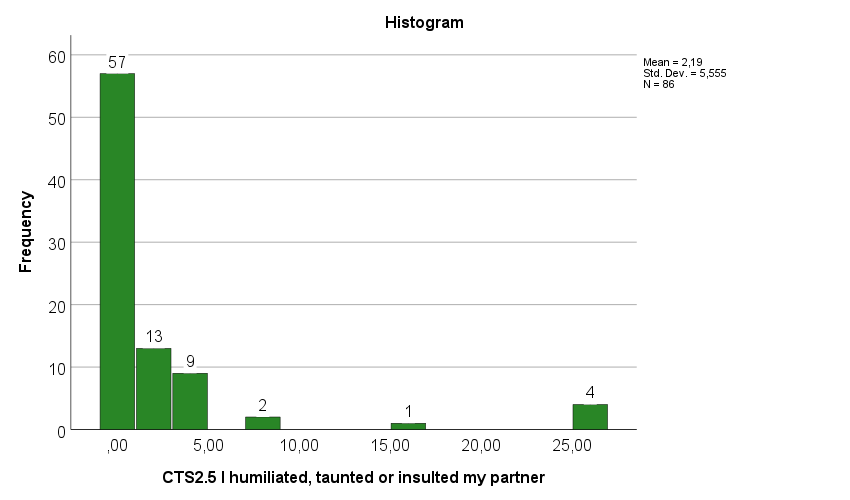


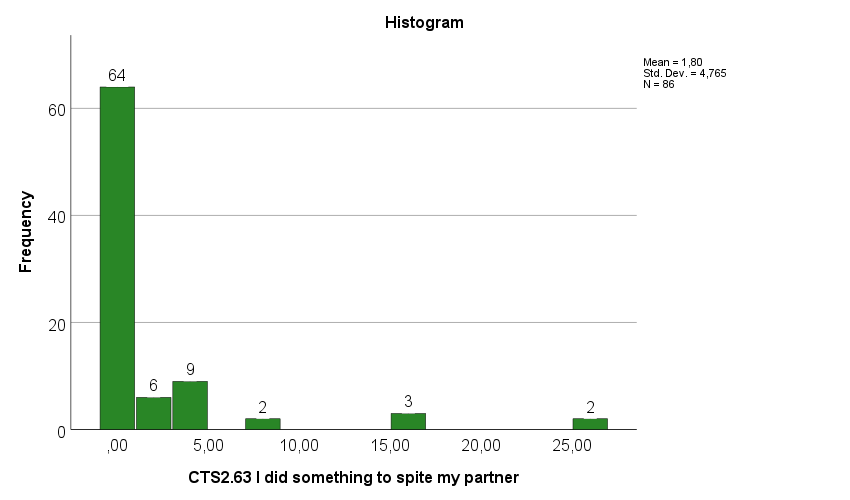


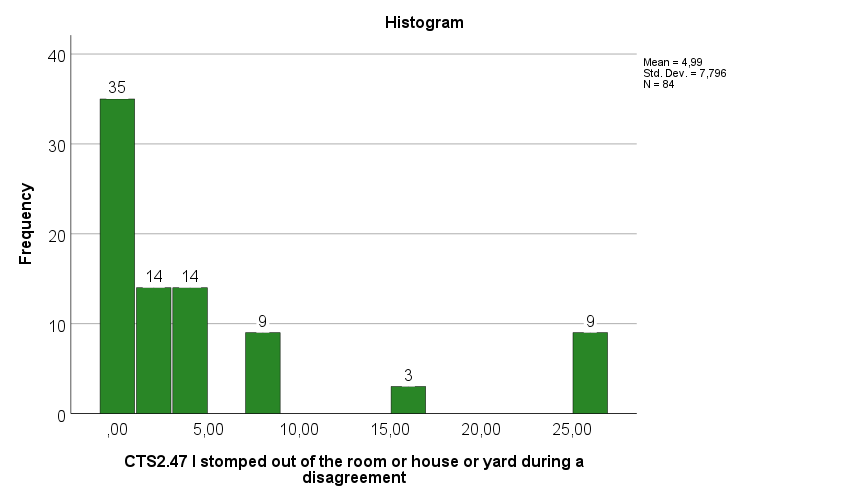


‘

**Appendix B**

**Cronbach’s α for Chronicity Subscales of the Revised Conflict Tactics Scale**

| Subscale | IPV victimization | IPV perpetration |
| --- | --- | --- |
| Psychological Aggression |  |  |
| Total Score | .612 | .638 |
| Minor | .188 | .442 |
| Severe | .620 | .508 |
| Physical Assault |  |  |
| Total Score | .911 | .624 |
| Minor | .826 | .600 |
| Severe | .865 | .266 |
| Sexual Coercion |  |  |
| Total Score | .900 | - |
| Minor | Only one item | Only one item |
| Severe | .843 | - |
| Injury |  |  |
| Total Score | .787 | .304 |
| Minor | .807 | .083 |
| Severe | .677 | .965 |

*Note.* For perpetration of sexual coercion, there were no scores other than 0. Thus, Cronbach’s α was not calculated.
